# Epidemiological Data, Clinical Signs, Therapy and Outcome Evaluation in Dogs with Syringomyelia of Different Etiology

**DOI:** 10.3390/vetsci12040376

**Published:** 2025-04-17

**Authors:** Rania D. Baka, Ioannis Savvas, Eirini Sarpekidou, George Kazakos, Zoe Polizopoulou

**Affiliations:** 1 Diagnostic Laboratory, School of Veterinary Medicine, Faculty of Health Sciences, Aristotle University of Thessaloniki, PC 54627 Thessaloniki, Greece; poliz@vet.auth.gr; 2 Companion Animals Clinic, School of Veterinary Medicine, Faculty of Health Sciences, Aristotle University of Thessaloniki, PC 54627 Thessaloniki, Greece; isavas@vet.auth.gr (I.S.); e.sarpekidou@gmail.com (E.S.); gkdvm@vet.auth.gr (G.K.)

**Keywords:** brain disease, Chiari-like malformation, dogs, outcome, skull disorder, survival, syringomyelia

## Abstract

Syringomyelia detected in both animals and humans may cause a variable degree of discomfort and its etiology is commonly unidentified. The aim of this study was to compare the outcome in dogs with those having syringomyelia of different etiology. Dogs with syringomyelia were subdivided into two groups: syringomyelia associated with Chiari-like malformation (S-CLM) (15 dogs) and syringomyelia of other etiology (SOA) (15 dogs). Age onset of S-CLM clinical signs was earlier compared to SOA (mean S-CLM and SOA values: 50.53 and 97.6 months, respectively, *p* = 0.021). Two neurological dysfunction scoring systems alongside nociception values were lower in SOA compared to S-CLM (mean values for neurological dysfunction scoring system SOA and S-CLM: 5.87 and 4.2, respectively, *p* = 0.032) (mean values for nociception SOA and S-CLM: 20.97 and 10.03, respectively, *p* = 0.03). Symptomatic therapy included combinations of corticosteroids, gabapentin (10/15, 66.6%) in S-CLM and NSAID +/− gabapentin (8/15, 53.3% and 9/15, 60%, respectively) in SOA dogs. Eight S-CLM dogs (53.4%) improved with symptomatic therapy and eleven were still alive; however, most SOA dogs (9/15, 73.4%) died/were euthanized by the end of this study. SOA dogs demonstrated more severe neurological signs compared to S-CLM, although the outcome between the two groups was not associated (*p* = 0.211).

## Simple Summary

Syringomyelia may cause discomfort, pain and neurological deficits in both humans and animals. In dogs, its etiology cannot be easily identified except for Chiari-like malformation, which can contribute to syringomyelia in small-breed dogs. The current study evaluated the epidemiological data, clinical signs, therapy and outcome in dogs diagnosed with syringomyelia associated with Chiari-like malformation (S-CLM) and in dogs whose etiology could not be identified despite thorough diagnostic investigation (SOA). Age onsets of clinical signs appeared earlier in S-CLM dogs compared to those with SOA. SOA dogs demonstrated more severe neurological signs compared to those with S-CLM, assessed by two neurological dysfunction scoring systems. The outcome was negatively affected in SOA dogs since either death occurred or euthanasia was chosen in SOA patients by the end of this study. Syringomyelia of undetermined/unknown etiology may negatively impact quality of life and outcome in dogs despite medical therapy being administered. The current study may contribute to the estimation of the prognosis in dogs diagnosed with syringomyelia of different etiology.

## 1. Introduction

Syringomyelia is the progressive development of a fluid-filled cavitation (“syrinx”) within the cervical, thoracic and, occasionally, lumbar spinal cord parenchyma, most often affecting the dorsal horn, resulting in sensory processing abnormalities due to disruption of normal pathways [1,2], caused by the abnormal flow of cerebrospinal fluid (CSF) [3]. The abnormal dilatation of the central canal, with an intact ependymal layer, is referred to as hydromyelia and is regarded as the preliminary stage of syringomyelia [4]. Syringomyelia is a painful condition, more commonly detected in toy breeds including Cavalier King Charles Spaniel (CKCS) than other breeds [5]. In these toy breeds, syringomyelia is associated with a specific skull malformation called Chiari-like malformation (CLM) [5]. The Cavalier King Charles Spaniel (CKCS) is predisposed and the presence of syringomyelia is correlated with a more extreme CLM [6]. In particular, CLM is a complex developmental malformation of the skull and cranial cervical vertebrae, characterized by rostro-caudal bony abnormality leading to conformational changes and overcrowding of the caudal brain and cervical spinal cord, particularly in the craniocervical junction [7]. Apart from abnormalities observed in the craniocervical junction (CLM, atlanto-occipital overlapping, atlanto-axial instability), other problems such as achondroplasia, spinal diverticulum, intervertebral disc disease, spinal canal stenosis and kyphosis have been associated with syringomyelia [8,9,10,11,12].

Syringomyelia may cause neurological symptoms, but it can often be asymptomatic [3,13,14]. Dogs with syringomyelia associated with Chiari-like malformation may be presented with neuropathic pain, cervical myelopathy and brainstem, cerebellar or vestibular dysfunction [15]. Cervical hyperesthesia may be noted on spinal palpation, although pain can be nonspecific, intermittent and spontaneous (not caused by an obvious stimulus) or may be manifested by behavioral changes often identified by the owners [16,17]. Vocalization, change in activity, reduced exercise tolerance, lethargy and changes in emotional state (e.g., timid, anxious, withdrawn, aggressive animals) have been reported [18]. Head position during sleep may be indicative of pain relief and dog owners often report that their dogs tend to sleep with their heads in an elevated position since head flexion increases the length of cerebellar herniation, which causes discomfort [16]. Phantom scratching is a common and unique clinical sign identified in syringomyelia associated with CLM [17], and it is an indication of behavioral expression of allodynia or paresthesia, typically oriented towards one side of the neck, often correlating with lateralization of the syrinx [1,17,19]. Phantom scratching is differentiated from true pruritus because there is no contact of the animal’s paw with the skin [1,17]. The signs that are significantly associated with syrinx presence and width are phantom scratching and scratching or rubbing the ears and the head, scoliosis, postural deficits or weakness, as indicated by Rusbridge et al. (2018) [20]. As indicated in humans, syringomyelia may be detected during diagnostic imaging; however, the underlying cause of syrinx formation may not be determined [21,22].

Beyond phantom scratching, neurological examination of the dog with syringomyelia secondary to CLM may be normal or may reveal proprioceptive deficits, lower motor neuron signs in the thoracic limbs, ataxia or paresis [15,23]. Depending on the width and location of syringomyelia, clinical signs may vary widely [3]. The severity is often associated with large syringes; however, gait disturbance may be mild even in cases involving the entire spinal canal [24].

Therapeutic options for CLM and/or syringomyelia and syringomyelia of other etiology include medical and surgical management [25,26]. Medical management includes non-steroidal anti-inflammatory drugs (NSAID), drugs that reduce CSF production (omeprazole, cimetidine), corticosteroids, and antiepileptic drugs that have analgesic properties (gabapentin, pregabalin, topiramate) [25,27]. Gabapentin is widely used in veterinary patients with suspected neuropathic pain [3,28,29]. Topiramate’s use is limited [30,31] and it has been evaluated alone or combined with non-steroidal anti-inflammatory drugs (NSAID) [27]. Surgical therapy applied in CLM patients has only moderate success [32]. Despite surgical decompression of the craniocervical region, most dogs with CLM and syringomyelia improve; however, there is no syrinx resolution and all dogs continue to exhibit clinical signs compatible with neuropathic pain postoperatively [33,34,35,36]. In one-quarter to half of the dogs managed surgically, clinical signs reoccur after surgery [34,35,36]. In post-traumatic syringomyelia cases, surgical treatment could not guarantee symptomatic resolution or even prevention of further neurological loss [21].

Limited data are available regarding the long-term outcome of dogs with CLM combined or not with syringomyelia which underwent medical treatment [25]. Scoring systems for the severity of clinical signs have been proposed by other research groups to indicate, on a numerical scale, the progression of the disease [25,37,38]. Less than half of the patients indicated long-term improvement in post-traumatic syringomyelia [21].

The purpose of the current study was to present epidemiological data, clinical signs, therapy undertaken and long-term outcome in dogs exhibiting syringomyelia secondary to Chiari-like malformation (S-CLM) and syringomyelia of different etiology (SOA).

## 2. Materials and Methods

This retrospective study included medical records from canine patients that were admitted to the Companion Animal Clinic of the School of Veterinary Medicine, Faculty of Health Sciences, Aristotle University of Thessaloniki, from January 2018 to June 2022 and were diagnosed with syringomyelia. The major inclusion criterion for the study population was diagnosis of syringomyelia with MRI scan of the central nervous system. Dogs were subdivided into two groups based on the origin of syringomyelia: one group consisted of dogs with syringomyelia secondary to Chiari-like malformation (S-CLM) and the other group consisted of dogs with syringomyelia of other etiology (SOA).

Information retrieved from the medical records included epidemiological data (breed, age, gender, body weight, body score, living status—indoors/outdoors/both), historical data (presenting complaint, age onset of clinical signs, clinical signs duration and progression, previously administered medication and the subsequent response to treatment), and physical and neurological examination findings. All dogs received a neurological dysfunction score (two neurological dysfunction scoring systems were applied) and a pain assessment score (Glasgow pain scoring system for dogs) using respective scoring systems (Table 1) [37,39,40]. Pain caused by other (extraneural) conditions was an exclusion criterion for the study population. Treatment strategies (surgical decompression and/or medical management) were recorded. Follow-up was performed either via neurological re-assessment of the cases or via phone call for those who were unable to re-submit their dogs, and included the progression of signs and the outcome accompanied by a video of their dogs, sent electronically for neurological assessment of the case. For surviving dogs, the days that were neurologically stable (no signs of deterioration) were also recorded.

Comparisons were performed between the two groups regarding the age onset of clinical signs, the severity of neurological signs and pain. Outcome was also evaluated between the two groups to assess whether the etiology of syringomyelia may influence outcome and quality of life.

The neurological dysfunction scoring system developed for the purposes of the current study is presented in Table 1. Lower scores indicated a less severe neurological status. The scoring system for neurological dysfunction as described by Lewis and Olby (2017) [37], based on nociception evaluation, was used for comparison purposes during statistical analysis.

The body score of the dogs in both groups was assessed as a contributing factor affecting outcome. The five-scale body condition scoring system was used for the study population groups. For statistical analysis purposes, normal and underweight dogs were grouped together and overweight dogs were put into a separate group. Both neurological dysfunction scoring systems used in the population groups of the current study were assessed as contributing factors to the outcome. Age onset of symptoms of S-CLM was compared with age onset of SOA symptoms. Both neurological dysfunction scoring systems of S-CLM were compared with the scoring systems of SOA. The Glasgow Pain Scale scores of S-CLM were compared with those of SOA. Follow-up evidence as reflected by the days that S-CLM dogs were neurologically stable was compared with the follow-up days of neurological stability in SOA dogs.

Descriptive statistics were produced using SPSS 19.0. The Shapiro–Wilk (S-W) test for normality was used to examine whether the continuous variables followed the normal distribution. For the variables that follow the normal distribution, the *t*-test was used. For the variables that did not follow the normal distribution, the Mann–Whitney test was used. *p* ≤ 0.05 was set as the cut-off for significance.

## 3. Results

### 3.1. Epidemiological Data

Thirty dogs met the inclusion criteria and were enrolled in this study. Fifteen dogs were diagnosed with S-CLM and fifteen dogs with SOA. The epidemiological data of the study population dogs are recorded in Table 2. The median value of age for S-CLM was 6.5 years (min. value 1 year, max. value 9 years) and 8 years (min. value 4 years, max. value 12 years) for SOA dogs. The median value of body weight for S-CLM was 6.5 kg (min. value 2.5 kg, max. value 14 kg) and 17.1 kg (min. value 2.75 kg, max. value 37 kg) for SOA dogs. The median value of age onset of the clinical signs for S-CLM was 54 months (min. value 2 months, max. value 108 months) and 96 months (min. value 42 months, max. value 144 months) for SOA dogs. The median value for duration of symptoms for S-CLM was 90 days (min. value 1 day, max. value 1825 days) and 30 days (min. value 1 day, max. value 150 days) for SOA dogs.

### 3.2. Neurological Examination Findings

Table 3 summarizes the neurological examination findings of the two groups of dogs.

Muscle atrophy was observed in one S-CLM dog and was localized in the forelimbs. The same dog exhibited epileptic seizures, compulsive behavior and was depressed. Regarding cranial nerve deficits, bilateral mydriasis was seen in one S-CLM dog and two dogs displayed anisocoria, one dog with absent and one dog with decreased (right-sided) papillary light reflex. Neck movement was abnormal in 8/15 (53.3%) of S-CLM dogs. In these eight dogs with abnormal neck position and movement, there was cervical hyperesthesia and pain in four (50%) dogs, neck extension in two (25%) dogs, resistance in changing neck direction movements in one (12.2%) dog and neck scratching in one (12.2%) dog. The pain evaluated with the Glasgow Pain Scale of the two groups is recorded in Table 4.

For SOA dogs, generalized muscle atrophy existed in one dog. Six SOA dogs were admitted for lateral decumbency and all of them had muscle hypertonicity. One dog presented with left anisocoria (left mydriasis) with normal response to papillary light reflex. There was evidence of spinal pain in six dogs (40%). Three dogs (20%) had loss of nociception (grade V), while one dog presented with hypoesthesia (6.6%) and two dogs had hyperesthesia (13.3%). Micturition disorders were recorded in thirteen dogs (73.3%):five dogs had upper motor neuron urinary disorders (distended urinary bladder) and eight dogs had lower motor neuron urinary disorders (small-sized urinary bladder, easily expressed upon manipulation). Four dogs showed neck movement disorders: one dog showed pain (6.6%), two dogs showed pain when the neck was flexed (13.3%) and one dog demonstrated extension resistance (6.6%).

Table 5 summarizes the neuroanatomical localization of the study population.

### 3.3. Management

Only two cases (13.3%) underwent surgical decompression of the caudal fossa. The other 10 S-CLM dogs underwent medical management with corticosteroids along with gabapentin (10/15, 66.6%). One of the dogs was managed medically and received opioids (tramadol and/or butorphanol; Torbutrol, Zoetis, Parsippany-Troy Hills, NJ, USA) (along with corticosteroids and gabapentin). All dogs in the SOA group underwent medical management. The underlying etiology for syringomyelia could not be determined in this group. Medical management of SOA dogs included corticosteroids (3/15, 20%), NSAID (firocoxib; Previcox, MERIAL, Mumbai, India) (8/15, 53.3%), opioids (tramadol and/or butorphanol; Torbutrol, Zoetis) (5/15, 33.3%) and gabapentin (9/15, 60%) administration. All SOA dogs followed a physical rehabilitation program alongside their medical management.

### 3.4. Follow-Up

During follow-up of S-CLM dogs, eight dogs improved, two were stable and five deteriorated. Eleven dogs were still alive by the end of the study period. Three dogs were either euthanized or died due to S-CLM, and one dog died because of chronic kidney disease. The median value in clinical sign changes (either improvement or deterioration) was 120 days for S-CLM dogs (min. value 7 days, max. value 1825 days). Five SOA dogs improved (33.3%), two were stable (13.3%) and six deteriorated (40%) during follow-up. By the end of this study, six dogs were still alive (26.6%) and nine dogs either died or were euthanized (73.3%). The median value in clinical sign changes (either improvement or deterioration) was 30 days (min. value 0 days, max. value 1825 days) for SOA dogs.

### 3.5. Body Score Associated with Outcome

Neither S-CLM nor SOA showed a significant association with patients’ outcome (*p* = 0.462, *p* = 0.185, respectively).

### 3.6. Neurological Dysfunction Score Associated with Outcome

Neither S-CLM nor SOA demonstrated a significant association with patients’ outcome (*p* = 0.569, *p* = 0.109, respectively).

### 3.7. Comparisons Between S-CLM and SOA Dogs

#### 3.7.1. Age Onset of Clinical Signs

The mean age onset of clinical signs for the S-CLM dogs was 50.53 months (4.2 years), while the mean age onset of clinical signs for the SOA dogs was 97.6 months (8.1 years). The results from the statistical analysis (*t*-test) demonstrated that there was a significant difference between S-CLM and SOA dogs, and symptoms appear earlier in S-CLM dogs than SOA dogs (*p* = 0.021).

#### 3.7.2. Neurological Dysfunction Score

The mean value for the neurological dysfunction test for S-CLM dogs was 4.2, while the mean value for SOA dogs was 5.87. The *t*-test results showed a significant difference in the neurological dysfunction scoring system between the two groups of dogs (S-CLM and SOA dogs). In particular, the neurological dysfunction score in S-CLM dogs was lower than for SOA dogs (*p* = 0.032), indicating that neurological signs of S-CLM dogs were not as severe as those of SOA dogs.

#### 3.7.3. Nociception Scoring System

The mean values for the nociception scoring system for S-CLM and SOA dogs were 1.8 and 3.87, respectively. The Mann–Whitney test (variables did not follow the normal distribution) indicated that SOA dogs had significantly higher nociception compared to S-CLM dogs (*p* = 0.04).

#### 3.7.4. Glasgow Pain Scale

Pain scoring between the two groups was also evaluated with the Glasgow Pain Scale. The mean values for S-CLM and SOA dogs were 6.13 and 4.07, respectively. The Mann–Whitney test demonstrated that there was no statistically significant difference between the Glasgow Pain Scale scores of the two groups (*p* = 0.167).

#### 3.7.5. Outcome

During follow-up, eight S-CLM dogs improved, two were stable and five deteriorated. In the SOA group, five dogs improved, three dogs were stable and seven dogs deteriorated. The outcome between S-CLM and SOA dogs did not show any significant difference (*p* = 0.211).

## 4. Discussion

The purpose of this study was to present and compare data in dogs with syringomyelia of different etiology. Much research has been published regarding syringomyelia secondary to Chiari-like malformation, especially in CKCS, but limited data are available regarding cases of different (undefined) etiology.

The results of this study demonstrated that age onset of clinical signs was significantly earlier in S-CLM dogs (median value 6.5 years) compared to SOA dogs (median value 8 years). This is quite logical since CLM is a congenital malformation that exists from the animal’s birth and progressively deteriorates. Other studies demonstrate an earlier onset of clinical signs in dogs with CLM (from 6 months to 2 years) [1]. The mean value of age onset of clinical signs from dogs with S-CLM was 2.2 years in another study [36]. In contrast, another study indicated the increased incidence of diagnosing symptomatic S-CLM in dogs over five years of age compared to dogs less than one year of age [41]. Since CLM is an unpredictable but progressive disease, clinical signs may become apparent at different ages. Moreover, age onset of clinical signs was provided by the owners during history-taking; therefore, some earlier indications of disease onset (e.g., behavioral changes due to pain) may be misinterpreted. Therefore, there are variations in age onset of clinical signs among different studies. Although etiology of syringomyelia (not associated with CLM) may still be unknown despite a thorough diagnostic imaging investigation, other causes that may affect the spinal cord and the dynamics of CSF may be related to degenerative, vascular or idiosyncratic properties. Syringomyelia is considered to be a chronic spinal cord injury [42]. As a spinal cord injury, it is associated not only with damage to the nerve tissue–CSF barrier but also with damage to the nerve tissue–blood barrier [43]. The pre-oxidative and antioxidant processes that occur in the central nervous system may reflect the pathogenetic mechanisms of syringomyelia formation but the molecular pathways associated with syringomyelia formation need better clarification [26]. More established knowledge in pathogenetic or etiologic mechanisms of the disease may reveal markers associated with age. In humans, syringomyelia is diagnosed in young adults, and apart from CLM, spinal cord trauma and arachnoiditis are correlated with syringomyelia [44].

The S-CLM dogs in this study were small-breed dogs (mean body weight 6.5 kg), while the SOA dogs were medium-sized dogs (mean body weight 17.1 kg). The small-sized S-CLM dogs is aligned with a previous study by Rusbridge et al. (2018) [20] that indicated CLM in dogs of less than 10 kg. CLM is found in dogs with a specific skull anatomy, most commonly found in small-breed dogs [45]. A study performed by Sanchis-Mora et al. (2016) [41] indicated breed (small-breed dogs) as a risk factor for diagnosis of S-CLM. However, there are no data available regarding body weight for dogs with syringomyelia of other (undefined) etiology. This may be a finding to be further evaluated in the future in an attempt to determine whether increased body size of a dog may be a risk factor for syringomyelia formation. Body score in neither of the two groups was associated with patient outcome. We speculated that increased body score probably increases pain, especially during physiotherapeutic manipulation of SOA dogs (which were larger-sized dogs), and subsequently decreases quality of life. However, the statistical analysis failed to reveal any associations between the two variables. It is probable thata larger number of study population dogs may be needed to adequately evaluate this variable.

While the neuroanatomical localization in the S-CLM group was focused on cervical and intracranial disease, in the SOA group, there was an almost equal number of cases presenting with cervical (8/15) and thoracolumbar (7/15) myelopathies with syrinx formation. There is no available research supporting the high incidence of syringomyelia in these two parts of the spinal cord. However, based on human experimental and theoretical remodeling studies in the literature, syrinx formation and enlargement may result from an imbalance between fluid inflow and outflow [21]. Since CSF may normally circulate from the subarachnoid space into the spinal cord extracellular space, changes in subarachnoid space compliance CSF fluid or pressure dynamics from arachnoid adhesions, spinal stenosis or cord compression may increase fluid inflow, leading to syrinx enlargement [46,47]. CSF dynamics may be associated with syrinx formation in the cervical spinal cord since the cervical part is the first anatomical region between the brain and spinal cord where CSF flows from larger to smaller cavities when there is no detectable lesion that may obstruct CSF flow (self-evidence). The intercapital ligament that lacks caudal thoracic spinal cord stabilization may reflect the incidence of spinal trauma and syrinx formation after disc extrusion in non-small-breed dogs.

Syringomyelia is a quite unpredictable disease entity: clinical signs may vary from asymptomatic cases to severe pain and neurological deficits, while symptoms severity may correlate with syrinx size in both dogs and humans [24,48]. Both neurological dysfunction scoring systems utilized to numerically quantify qualitative variables (neurological signs; nociception scoring system and neurological dysfunction scoring system developed for current study purposes) indicated statistically significant differences between S-CLM and SOA dogs. In particular, both scoring systems indicated lower values for S-CLM dogs compared to SOA dogs. The higher the value of the scoring system was, the worse the neurological dysfunction the dog exhibited. Therefore, SOA dogs were more severely affected compared to S-CLM dogs. To the authors’ knowledge, there is no published research comparing S-CLM and syringomyelia of undefined etiology in dogs. In humans, it is indicated that most patients demonstrated slow progression of symptoms and signs, while rapid deterioration was recorded after myelography or due to syrinx hemorrhage from the cavity wall vessels, and other patients remained asymptomatic for more than 10 years [49,50,51,52]. Since syringomyelia is a progressive and unpredictable disease, the neurological dysfunction scoring systems that have been presented solely indicate the neurological status of the patient at the time of the examination and its utility is focused on repetitive neurological examinations of the patients over time to assess progression. However, SOA dogs were admitted to the clinic earlier than S-CLM dogs (mean values for symptomatology duration: 90 days for S-CLM dogs and 30 days for SOA dogs). The lower neurological dysfunction scoring system of SOA dogs with the shorted symptomatology duration may demonstrate that syringomyelia of undefined etiology may indeed cause more severe neurological dysfunction to dogs compared to CLM, which may exhibit a more gentle progression over time.

Apart from two dogs in the S-CLM group that were managed surgically, all other dogs underwent medical management focusing on pain control and improving quality of life rather than diminishing syrinx size or caudal brain decompression. Seven S-CLM dogs underwent combination therapy (including NSAID/corticosteroids and antiepileptic medication which included gabapentin) and two dogs underwent gabapentin monotherapy. Despite only two dogs having undergone surgical decompression of the caudal fossa, one-third of the S-CLM dogs deteriorated, while ten either improved or were stable, indicating a satisfying management of the majority of cases, albeit not statistically significant. Previous published research indicated that medical management can improve symptoms and quality of life in S-CLM in dogs that were not managed surgically [27,38]. None of the SOA dogs were managed surgically. Due to the undefined etiology of syringomyelia in dogs, surgical management is only applied in syringomyelia secondary to Chiari malformation [53]. However, in humans, a few surgical procedures have been found to diminish syrinx size, albeit all of them have been incriminated for high recurrence rates (>92% at 3 years), and there is a need for additional surgical procedures and frequent follow-up consultations [12,54]. All SOA dogs were medically managed with NSAID/corticosteroids, and/or gabapentin and/or opioids.

Although more dogs belonging to the S-CLM group either improved or remained neurologically stable (compared to SOA dogs), outcome and survival time did not show any significance between the two groups. Probably, the low number of cases was responsible for such a result. The median value of the period of time that S-CLM dogs did not show any signs of deterioration or even improvement was 120 days compared to the shorter period of time for SOA dogs (30 days). We may speculate that syringomyelia secondary to Chiari malformation may have a more favorable outcome with better quality of life compared to syringomyelia of other (undefined) etiology when dogs were managed medically. Unfortunately, this statement was not significant. Other future studies are required to evaluate medical management together with outcome in dogs with syringomyelia of other (undefined) etiology.

There were limitations in the current study which included its retrospective nature, with some missing data. The low number of cases may be responsible for the poor associations and correlations in the statistical analysis between the two groups. Although the numerical scoring system is a practical tool to quantify qualitative parameters (e.g., severity of clinical signs), it is not a reliable tool when comparing dogs with different neurological status modalities. Although syringomyelia was the diagnosis in both dog groups, S-CLM consisted of dogs with the diagnosis of syringomyelia secondary to CLM. However, in SOA, syringomyelia’s primary disease could not be determined. Therefore, the authors indicate that the proposed scoring system could be utilized in patients with the same diagnosis to compare disease severity and other parameters that may be associated with prognosis and outcome (mostly for research purposes). Data regarding outcome were collected either by physical examination and history-taking from the dog owner during consultation or via phone call for those cases that could not be admitted to the clinic. During phone calls, dog owners responded to questions regarding progression of the disease, response to therapy and overall quality of life, which is quite subjective although sometimes can be reliable data for the long-term outcome of the patients.

## 5. Conclusions

Clinical signs due to S-CLM may appear earlier compared to SOA in a dog’s lifespan. Small-breed dogs commonly exhibited S-CLM, while medium-sized dogs were diagnosed with SOA. The body score of the dogs was not associated with the outcome. SOA dogs were more severely affected compared to S-CLM dogs. Medical management can improve symptoms and quality of life in S-CLM dogs.

## Figures and Tables

**Table 1:** Numerical scoring system for neurological dysfunction in dogs.

Parameter Examined	Numerical Score and Definition
Mental status	0—normal, 1—confusion, 2—depression, 3—stupor, 4—coma
Cognitive function	0—normal, 1—abnormal
Seizures	0—no seizures identified 1—seizures identified during historytaking or during examination
Behavior	0—normal, 1—abnormal
Overall neurological assessment (after postural reaction, spinal reflexes, standing, walking, nociception evaluation) was performed	1—spinal hyperesthesia, 2—ambulatory paresis, 3—nonambulatory paresis, 4—paralysis with intact nociception, 5—paralysis without nociception
Cranial nerves: facial symmetry	0—normal, 1—abnormal
Cranial nerves: palpebral reflex	0—normal, 1—abnormal on one side, 2—abnormal on both sides
Oculovestibular	0—normal, 1—abnormal on one side, 2—abnormal on both sides
Gag reflex	0—normal, 1—abnormal
Tongue	0—normal, 1—abnormal
Menace response	0—normal, 1—abnormal in one eye, 2—abnormal in both eyes
Nasal stimulation	0—normal, 1—abnormal on one side, 2—abnormal on both sides
Pupil size	0—normal, 1—anisocoria, 2—mydriasis/miosis in both eyes
Nystagmus	0—no nystagmus present, 1—positional nystagmus, 2—spontaneous nystagmus
Strabismus	0—no strabismus present, 1—one eye, 2—both eyes, +1—positional strabismus, +2—permanent strabismus
Voluntary urination	0—normal, 1—abnormal
Spinal pain	0—no, 1—yes
Neck movement	0—normal, 1—abnormal

**Table 2:** Epidemiological data of the study population dogs.

	Syringomyelia Associated with Chiari-like Malformation (S-CLM)		Syringomyelia of Other Etiology (SOA)	
	Number (Total 15)	Percentage (%)	Number (Total 15)	Percentage (%)
Breed				
CKCS	7	46.6	0	
Maltese, Shih-tzu, Jack Russell Terrier, Yorkshire terrier	1 *	6.6	0	
Mixed breed	1	6.6	5	33.3
French bulldog	1	6.6	2	13.3
Pinscher	1	6.6	1	6.6
Chihuahua	1	6.6	1	6.6
Boxer, Belgian Tervuren, Miniature Poodle, Spitz, Labrador Retriever, Golden Retriever	0		1 *	6.6
Gender				
Female	7	46.6	7	46.6
Male	8	53.4	8	53.4
Body score				
2/5	0		2	13.3
3/5	9	60	10	66.6
4/5	6	40	4	13.3
5/5	0		1	6.6
Living conditions				
Indoors	14	93.3	11	73.3
Outdoors	0	0	3	20
Both	1	6.66	1	6.6
Cause for admission				
Pain	5	33.3	6	40
Phantom scratch	3	20	0	
Ataxia	3	20	0	
Paraplegia	0		5	33.3
Paraparesis	0		3	20
Tetraparesis	1	6.6	1	6.6
Tetraplegia	1	6.6	4	26.6
Seizures	1	6.6	1	6.6
Other	3	20	0	
Progression/Stability of symptoms				
Progression	9	60	12	80
Stability	6	40	3	20
Received medication when admitted				
Yes	13	86.66	12	80
No	2	13.33	3	20
Medication administered				
Corticosteroids monotherapy	2	13.33	5	33.3
NSAID monotherapy	1	6.66	5	33.3
Antiepileptic medication	2	13.33	0	
Combination	7	46.66	2	13.3
Responded to medication administered				
Yes	6	46.15	3	25
No	7	53.85	9	75

* Number 1 indicates1dog of each breed.

**Table 3:** Neurological examination findings of the S-CLM and SOA dogs.

	Syringomyelia Associated with Chiari-Like Malformation (S-CLM)		Syringomyelia of Other Etiology (SOA)	
	Number (Total 15)	Percentage (%)	Number (Total 15)	Percentage (%)
Level of consciousness				
Normal	13	86.6	11	73.3
Abnormal	2	13.3	4	26.6
Cognitive function				
Normal	13	86.6	15	100
Abnormal	2	13.3	0	
Seizures				
Apparent	1	6.6	1	6.6
Absence	14	93.3	14	93.3
Behavior				
Normal	14	93.3	15	100
Abnormal	1	6.6	0	
Muscle atrophy				
Absent	14	93.3	14	93.3
Apparent	1	6.6	1	6.6
Head Position				
Normal	12	80	13	86.6
Abnormal	3	20	2	13.3
Walking disorders				
Absent	10	66.6	1	6.6
Hypermetria	0		1	6.6
Paraparesis	1	6.6	2	13.3
Paraplegia			5	33.3
Monoparesis	1	6.6	0	
Tetraparesis	1	6.6	0	
Tetraplegia	1	6.6	6	40
Ataxia				
Absent	10	66.6	6	73.3
Present	5	33.3	4	26.6
Spinal pain				
Absent	9	60	9	40
Present	6	40	6	60

**Table 4:** Glasgow Pain Scale results of the study population dogs.

	Syringomyelia Associated with Chiari-Like Malformation (S-CLM)		Syringomyelia of Other Etiology (SOA)	
	Number (Total 15)	Percentage (%)	Number (Total 15)	Percentage (%)
Glasgow Pain Scale				
<6	7	46.6	10	66.6
>6	8	53.3	5	33.3

**Table 5:** Neuroanatomical localization of the study population.

	Syringomyelia Associated with Chiari-Like Malformation (S-CLM)		Syringomyelia of Other Etiology (SOA)	
	Number (Total 15)	Percentage (%)	Number (Total 15)	Percentage (%)
Anatomical localization				
Cervical	10	66.6	6	40
Cervical with cerebellum involvement	3	20	0	0
Cervical with brainstem involvement	1	6.6	1	6.6
Cervicothoracic	1	6.6	1	6.6
Thoracolumbar	0	0	6	40
Lumbosacral	0	0	1	6.6

## Data Availability

Data are not available due to privacy reconstructions.

## Abbreviations

The following abbreviations are used in this manuscript:

CKCS	Cavalier King Charles Spaniel
CLM	Chiari-like malformation
CSF	Cerebrospinal fluid
MRI	Magnetic resonance imaging
NSAID	Non-steroidal anti-inflammatory drugs
S-CLM	Syringomyelia associated with Chiari-like malformation
SOA	Syringomyelia of other etiology
